# Prevalence of JP2 and Non-JP2 Genotypes of *Aggregatibacter actinomycetemcomitans* and Oral Hygiene Practice of Kenyan Adolescents in Maasai Mara

**DOI:** 10.3390/pathogens10040488

**Published:** 2021-04-17

**Authors:** Dorte Haubek, Tonnie Mulli, Arthur Kemoli, Mark Lindholm, Hans Gjørup, Marie-Louise Milvang Nørregaard, Anders Johansson

**Affiliations:** 1 Section for Paediatric Dentistry, Department of Dentistry and Oral Health, Health, Aarhus University, DK-8000 Aarhus, Denmark; dorte.haubek@dent.au.dk (D.H.); milvang@dent.au.dk (M.-L.M.N.); 2 Department of Periodontology, Community and Preventive Dentistry, University of Nairobi, P.O. Box 34848, Nairobi 00100, Kenya; mullitonnie@yahoo.com; 3 Department of Paediatric Dentistry and Orthodontics, University of Nairobi, P.O. Box 34848, Nairobi 00100, Kenya; musakulu@gmail.com; 4 Division for Oral Microbiology, Odontology, Umeå University, 901-87 Umeå, Sweden; mark.lindholm@umu.se; 5 Center for Oral Health in Rare Diseases, Department of Maxillofacial Surgery, Aarhus University Hospital, DK-8200 Aarhus N, Denmark; hangjo@rm.dk; 6 Molecular Periodontology, Odontology, Umeå University, 901-87 Umeå, Sweden

**Keywords:** JP2 genotype, dissemination, tooth brushing, leukotoxin, chewing stick

## Abstract

*Aggregatibacter actinomycetemcomitans* is implicated in the etiology of periodontitis that affects adolescents. The monitoring and mapping of the geographic dissemination pattern of JP2 and non-JP2 genotypes of *A. actinomycetemcomitans* are of interest. In Africa, the highly leukotoxic JP2 genotype is known to be prevalent, particularly in north-west Africa. The aims of this study were to determine the prevalence of JP2 and non-JP2 genotypes and investigate the oral hygiene practices among adolescents living in Maasai Mara, Kenya. A total of 284 adolescents (mean age: 15.0 yrs; SD 1.1) were interviewed regarding their age, gender, medical history, and oral hygiene practice, and the number of teeth present was recorded. One subgingival pooled plaque sample from all the first molars of each participant was analyzed by conventional PCR. The mean number of permanent teeth present was 27.9 (SD: 2.0; range: 22–32; 95% CI: 27.7–28.1). Sixteen (5.6%) and two (0.7%) adolescents were positive for non-JP2 and JP2 genotypes, respectively. For the vast majority of the adolescents, the use of a toothbrush (99.3%) and toothpaste (80.1%), as well as some kind of toothpick (>60.2%), were part of their oral hygiene practice, with dental floss (0.4%) and/or mouth rinses (0.4%) rarely being used. We have, for the first time, identified Kenyan adolescents colonized with the JP2 genotype. The prevalence of the JP2 genotype of *A. actinomycetemcomitans* is low, a possible indicator that spreading through human migration from North and West Africa to East Africa is a rare occasion.

## 1. Introduction

Periodontitis frequently occurs in humans in African countries [1,2,3,4,5,6]. Among adolescents, much attention has been given to the oral bacterium, *Aggregatibacter actinomycetemcomitans*, due to its capacity to produce leukotoxin, an exotoxin that is able to kill important cells of the immune system and cause inflammation [7,8,9]. Among the numerous genotypes of *A. actinomycetemcomitans*, a highly leukotoxic genotype called JP2 has extensively been reported on [10,11,12]. Dahlén and co-workers [13] proposed that the JP2 genotype of *A. actinomycetemcomitans* is the best-known example of a specific bacterial subtype with implications for periodontal disease. This JP2 genotype is characterized by a 530 base pair (bp) deletion in the promoter region of the leukotoxin gene operon, suggested to be responsible for the enhanced leukotoxin expression [12]. In particular, the highly leukotoxic JP2 genotype of *A. actinomycetemcomitans* has been strongly associated with the initiation of periodontal attachment loss in adolescents [2,4,14].

Although other genotypes of *A. actinomycetemcomitans*, other bacterial species, factors related to the host response, and other types of risk factors are associated with increased risk of the development of periodontitis in adolescents [15,16,17,18,19], it is important from a global perspective to understand the pattern of dissemination of the highly leukotoxic JP2 genotype of *A. actinomycetemcomitans* to be able to ensure infection control and, thereby, prevent the development of severe periodontitis in young individuals [20].

The JP2 genotype, with an estimated origin dating around 2400 years ago [10], is found to be highly conserved based on analyses of JP2 genotype strains collected over more than 20 years from individuals with geographically different origins and living across a wide geographically area. The mapping of the geographic occurrence of the JP2 genotype has revealed that its colonization is greatly connected to individuals of African descent, but has spread to other parts of the world, including Europe and North and South America [2,4,8,21,22,23,24,25]. Today, human migration from one part of the world to another is a frequent event [26].

As a consequence of the geographical dissemination of the highly leukotoxic JP2 genotype of *A. actinomycetemcomitans*, European and North and South American periodontitis patients are occasionally also found to be carriers [12,16,17,27,28]. Thus, JP2 genotype carriers with severe periodontitis might appear geographically widespread, and therefore this topic is relevant to clinicians all over the world. Despite the finding that the JP2 genotype of *A. actinomycetemcomitans* is less frequent in continents other than Africa, both individuals and migrants from the African continent as well as individuals not originating from African countries can be carriers of the JP2 genotype of *A. actinomycetemcomitans* [12,16,27,28,29,30]. Except for clear signs of a high prevalence of the JP2 genotype in North and West African countries, the dissemination of the JP2 genotype of *A. actinomycetemcomitans* to the remaining parts of the African continent is not known or has been very rarely examined and reported [31]. For example, in the East-African country Kenya, nothing is known concerning the potential presence of the highly leukotoxic JP2 genotype of *A. actinomycetemcomitans*. Interestingly, a genotype of *A. actinomycetemcomitans*, characterized by a 640 bp deletion in the promoter region of the leukotoxin gene operon and high leukotoxicity according to results obtained by the use of a leukotoxicity assay [17], has been detected in one individual living in Sweden with Ethiopian origin, yet nothing is known about its overall prevalence or geographic dissemination pattern [17,31]. Concerning the JP2 genotype of *A. actinomycetemcomitans*, our hypothesis is that it will not be found in the Kenyan adolescent population living in the fairly remote area of Kenya, called Maasai Mara.

The aims of the present study were to report on the prevalence of JP2 and non-JP2 genotypes of *A. actinomycetemcomitans* and oral hygiene practices among adolescents living in Maasai Mara, Kenya.

## 2. Results

### 2.1. Number of Maxillary and Mandibular Teeth Present in the Oral Cavity of Kenyan Adolescents

Among the 284 teenagers participating in the study, the overall mean number of permanent teeth present in the mouth was 27.9 (SD: 2.0; range: 22–32; 95% CI: 27.7–28.1). One teenager had only 11 permanent teeth and multiple primary teeth present (most likely due to delayed eruption) in the oral cavity, and this participant was therefore excluded from the calculation of the mean number of permanent teeth present. In Table 1, the complete distribution of clinically visible teeth as well as the absence of teeth in the upper and lower jaws according to tooth type is given.

### 2.2. Prevalence of JP2 and Non-JP2 Genotypes of A. actinomycetemcomitans Among Adolescents Living in Maasai Mara

**Figure 1:**
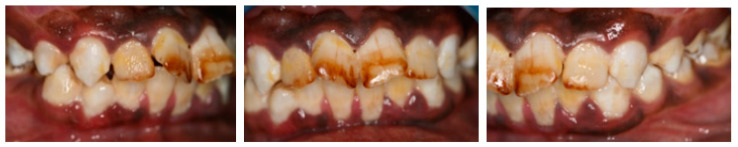
Clinical photos of an adolescent who tested positive for the JP2 genotype of *A.actinomycetemcomitans* (right view, frontal view, and left view, respectively). Red and swollen gingiva and irregular dental papillae are seen along the gum line.

A total of 16 (5.6%) adolescents among the 284 participants in the present study were found to be positive for the non-JP2 genotype of *A. actinomycetemcomitans*. Only two (0.7%) participants out of the 284 adolescents were found to be positive for the JP2 genotype of *A. actinomycetemcomitans* (Figure 1). Clinical photos of one of the cases positive for the JP2 genotype of *A. actinomycetemcomitans* are shown in Figure 1.

**Figure 2:**
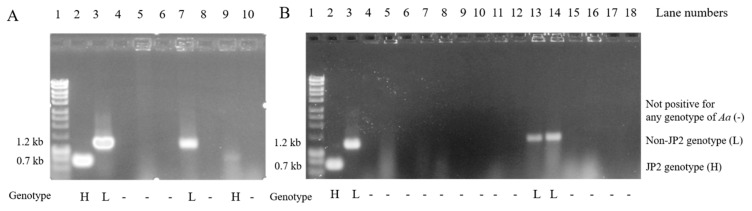
Agarose gel analysis of DNA fragments amplified by conventional polymerase chain reaction (PCR) directly from subgingival dental plaque samples. (**A**) Lane 1: a molecular weight marker illustrating sizes of the PCR-amplified DNA fragments; lane 2: a highly leukotoxic (H) reference strains of JP2 genotype of *A. actinomycetemcomitans* strain, HK921; lane 3: a low leukotoxic (L) reference strains of the non-JP2 genotype of *A. actinomycetemcomitans* strain, HK1605; lanes 4–10 illustrate findings in dental plaque samples from Kenyan adolescents, among whom the subject in lane 7 was positive for the non-JP2 genotype of *A. actinomycetemcomitans* and the subject in lane 9 was positive for the JP2 genotype of *A. actinomycetemcomitans.* The sizes of the fragments can be assessed according to the molecular weight marker in lane 1. The *A. actinomycetemcomitans* strains were examined for the occurrence of a 530 bp deletion in the promoter region of the leukotoxin gene (H), or not having this 530 bp deletion, being a non-JP2 genotype (L), thus marked by (H) or (L). If there is no band in the lane, the sample was not positive for any *A. actinomycetemcomitans* strains (-), and (**B**) illustrates in lanes 13 and 14 two samples from the same patients (reproducibility samples), with both samples from the same patient being positive for the non-JP2 variant of *A. actinomycetemcomitans*. Lanes 15 and 16 are loaded also with two samples from the same patients (reproducibility samples), with both samples being negative for *A. actinomycetemcomitans*.

Concerning the reproducibility of the *A. actinomycetemcomitans* findings in the respective samples of dental plaque, 12 out of the 284 (4.2%) individuals were sampled twice, with two of them testing positive for the non-JP2 genotype of *A. actinomycetemcomitans* at both sampling occasions (one of the non-JP2 genotype positive cases is illustrated in lanes 13–14; see Figure 2B). The other 10 samples were negative for *A. actinomycetemcomitans* at both sampling occasions, irrespective of genotype.

### 2.3. Answers to Questions on Oral Hygiene Tools and Hygiene Practices Used

The answers to the specific question raised on oral hygiene practice to the 284 Kenyan adolescents living in Maasai Mara are summarized in Table 2.

For the vast majority of the adolescents, the use of a toothbrush (99.3%) and toothpaste (80.1%), as well as some kind of toothpick (also called chewing stick or Mswaki) (>60.2%), was a part of their oral hygiene practice, whereas the use of dental floss and/or mouth rinse was extremely rare (0.4% and 0.4%, respectively) (Table 2). The majority of the adolescents brushed their teeth every day (99.3%), the vast majority in the morning (95.4%), and very few (0.7%) answered that they never brushed their teeth. A total of 209 (73.6%) reported brushing their teeth two to three times per day, 67 (23.6%) reported brushing once per day, 6 (2.1%) adolescents reported brushing less than once per day, and only two (0.7%) answered that they never brushed their teeth. In addition, toothpaste with fluoride was very often reported to be used (>80%). About two thirds of them reported using other things while cleaning their teeth—for example, small branches from trees and leaves or chewing sticks (Mswaki) (72.2%).

## 3. Discussion

In the current study, we report, for the first time, findings on the highly leukotoxic JP2 genotype of *A. actinomycetemcomitans* in adolescents living in Kenya. The findings are, however, rare in the adolescent population living in Maasai Mara, Kenya (below 1% carriers), but it is now clear that the highly leukotoxic JP2 genotype of *A. actinomycetemcomitans* is also present in Kenya.

The present research project was carried out in the Mara North Conservancy in Maasai Mara, a rural area of Kenya, where the Maasai Mara National Reserve is situated. In this area, although human–wildlife interaction combined with the interaction with the tourists visiting the National Reserve can be challenging to the indigenous Maasai population living there, this population still maintains their traditional life. It is probable that access to more information through the internet, computers, and television might, over time, increasingly lead to behavioral and lifestyle changes in the population. In addition, changes in the oral microbiome of the Maasai population are likely to occur in the future due to increasing interaction with visitors in the Maasai Mara National Reserve, as well as when some members of the community travel outside Maasai Mara.

Concerning the oral health practices of the adolescents living in Maasai Mara, this is of interest, given the reports from previous studies carried out in African countries which show that unconventional oral hygiene tools may be used, such as branches from trees, leaves from bushes, or chewing sticks [32,33]. The use of important and conventional oral hygiene tools such as toothbrushes, toothpaste, and toothpicks was, however, high. The number using traditional oral hygiene tools, such as twigs from specific trees, was high, despite also using commercial conventional tools. In various populations around the world, people live under different life circumstances, with different resources available to them and with different risks for the development of periodontitis. To be at the forefront of a potential need for the development of new, efficient oral hygiene practices, we need to know about the current oral hygiene practices employed. We then might be able to recommend or even develop new oral hygiene tools and preventive strategies for the future that are acceptable to the focus population. For example, plant materials may contain beneficial plant extracts that exert an anti-leukotoxic potential [32,33,34,35]. Recently, proanthocyanidins and epigallocatechin gallate of plant origin have been found to neutralize the effect of the *A. actinomycetemcomitans* leukotoxin [35,36,37].

The participants in our study came from the Mara North Conservancy from part of the Maasai population. The present study sample represents the Maasai living in Maasai Mara only. The Maasai population with its semi-nomadic lifestyle differs greatly from the Kenyan population in general. Thus, the results obtained in the present study may differ from the potential data and, thereby, research findings based on other parts of the Kenyan population.

The clinical examinations were carried out in classrooms at the participating schools and the lighting was of varying quality. It is not known precisely how this may have affected the results. However, the results are expected to have been minimally affected, if affected at all, by the working conditions and the available natural light sources, given that the recording of teeth present in the oral cavity was not demanding in terms of the requirement of the light source. Hence, these available conditions were judged as sufficient for fulfilling the requirements of the present study.

Concerning the prevalence of the JP2 and non-JP2 genotypes of *A. actinomycetemcomitans*, the methodology applied in the present study has been used in several previously published studies [2,10,11,33,38]. As previously reported, the prevalence of the JP2 genotype of *A. actinomycetemcomitans* is high in North and West Africa (up to 13% of the adolescent population is colonized with the highly leukotoxic genotype of *A. actinomycetemcomitans*) [2]. Further, in a cohort consisting of 180 young African Americans with and without localized aggressive periodontitis (with 60 being localized aggressive periodontitis patients, 60 healthy siblings, and 60 unrelated health controls) in North Florida, the occurrence of JP2 genotype-positive subjects was evident. Altogether, 90 JP2 genotype-positive subjects (50%) were found in that study [14]. In comparison to such findings, the observations of the JP2 genotype in the present Maasai population can still be considered as very rare (0.7%). It is unknown if the low prevalence of the JP2 genotype of *A. actinomycetemcomitans* in Maasai Mara is unique or a common characteristic of the whole Kenyan population. However, the very low level of dissemination of the JP2 genotype of *A. actinomycetemcomitans* to the Maasai Mara region in Kenya might be associated with the very limited migration from North and West Africa to Maasai Mara in East Africa.

The present study shows for the first time that a few adolescents from Maasai Mara, Kenya, are colonized with the highly leukotoxic JP2 genotype of *A. actinomycetemcomitans*. The prevalence of the JP2 genotype of *A. actinomycetemcomitans* is, however, low. This indicates that the human migration of populations from North and West Africa to Maasai Mara in East Africa and, thereby, the potential risk of transfer of the JP2 genotype of *A. actinomycetemcomitans* through close interpersonal contacts, is most likely low. Regarding oral hygiene practices, while the majority of the adolescents used commercial toothbrushes and toothpaste, more than 60% reported using small branches from trees, leaves, or the local chewing sticks (Mswaki) as other means of maintaining oral hygiene.

## 4. Materials and Methods

### 4.1. Study Population

The study was conducted in Maasai Mara, more specifically in Mara North Conservancy, Kenya. The study population consisted of 284 Kenyan teenagers (mean age: 15.0; SD 1.1; range 14–18 years) recruited from the four primary and one mixed secondary schools in the Mara North Conservancy, Maasai Mara, Narok County of Kenya, as previously reported by Kemoli and coworkers [39]. The study population was representative of the Mara North Conservatory of Maasai Mara and consisted of healthy adolescents present at school on the day of the examination. None of the participants reported having smoked previously nor being an active smoker at the time of the study (one missing answer out of a total of 284 respondents). None of the participants reported having diabetes (missing answers from three out of 284 participants).

The study consisted of a face-to-face interview using structured questionnaires, a clinical examination to record the presence of participants’ teeth in the oral cavity, the collection of dental plaque sample(s) from mesial sites of all permanent first molars, and finally, the intra-oral photographing of the dentition.

### 4.2. Face-to-Face Interview

Structured questionnaires were used to collect data on age, gender, medical history, and the use of oral hygiene tools according to procedures previously described [39].

### 4.3. Recording of the Presence of Maxillary and Mandibular Teeth in the Mouth

The recording of the teeth present in the oral cavity was performed, and teeth were recorded as present when either partly or fully erupted. Oral examinations, including inter-rater reliability testing, were conducted as previously reported [39]. A procedure including the recall of the subjects for traditional intra-reliability evaluation was not possible due to the limited working time at the research site. As part of the data collection procedure, intraoral photographs were taken for the right, left, and frontal perspectives of the teeth in occlusion.

### 4.4. Procedure for Collecting and Analyzing Dental Plaque Samples

The collection of dental plaque samples for conventional polymerase chain reaction (PCR) analysis was carried out in all participants with the help of sterilized disposable mouth mirrors and tweezers. The subgingival plaque sample of the oral microbiota was collected with paper points (one separate, autoclaved package per participant, including four paper points, size 40; TopDent^®^, Väsby, Sweden) inserted subgingivally in the gingival crevice for 10–15 s at the mesial aspect of all first permanent molars. If one or more of these permanent first molars were not present, no sample from this/these quadrant(s) was included in the pooled plaque sample. The paper points from each patient were pooled into a tube containing 1 mL of 0.9% (weight/volume) NaCl.

Samples for the PCR detection of *A. actinomycetemcomitans* were analyzed at the Department of Biomedicine, Aarhus University, Denmark. Samples were processed and analyzed blindly by conventional PCR for the presence of the JP2 and non-JP2 genotypes of *A. actinomycetemcomitans*, as described previously [40]. Notably, the PCR detected the presence of the bacterium and distinguished between the JP2 and non-JP2 genotypes based on the distinct sizes of the amplicons because the JP2 genotype strains have a characteristic 530 bp deletion in the promoter of the leukotoxin operon.

### 4.5. Data analysis

The data collected were cleaned, coded, and entered into the computer, then analyzed with the use of SPSS 24 (Statistical Package for the Social Sciences, SPSS Inc., Chicago, IL, USA) and STATA 14.0 (StataCorp LLC, TX, USA).

## Figures and Tables

**Table 1:** The number of permanent and primary maxillary and mandibular teeth according to tooth type in 284 adolescents.

Tooth	Permanent Teeth Present	Permanent Teeth Absent		Primary Teeth Present	
Types	Bilateral	Bilateral	Unilateral	Bilateral	Unilateral
M3 max ^a^	88	170	24	-	-
M2 max	278	2	4	-	-
M1 max	279	0	5	-	-
P2 max	276	0	1	4	3
P1 max	279	0	4	1	0
C max	269	4	7	2	2
I2 max	274	6	3	0	1
I1 max	282	0	2	0	0
M3 mand ^a^	107	154	21	-	-
M2 mand	277	3	4	-	-
M1 mand	276	2	6	-	-
P2 mand	280	0	1	0	3
P1 mand	282	0	1	0	1
C mand	267	5	10	1	1
I2 mand	263	5	14	0	2
I1 mand	108	164	12	0	0

^a^ Missing data on two individuals; M: Molar; P: Premolar; C: Canine; I: Incisor; max: maxillary, meaning teeth in the upper jaw; mand: mandibular, meaning teeth in the lower jaw.

**Table 2:** Answers to questions from interviewing the 284 Kenyan adolescents on their oral hygiene practices.

Questions	Yes (%)	No (%)	Missing Data (%)
Do you have your own toothbrush?	254 (89.4)	28 (9.9)	2 (0.7)
Do you brush your teeth every day?	282 (99.3)	2 (0.7)	0 (0.0)
Do you brush in the morning?	271 (95.4)	12 (4.2)	1 (0.4)
Do you brush at lunchtime?	164 (57.7)	120 (42.2)	0 (0.0)
Do you brush in the evening?	179 (63.0)	105 (37.0)	0 (0.0)
Do you brush at other times of the day?	6 (2.1)	278 (97.9)	0 (0.0)
Do you use tooth floss?	1 (0.4)	263 (92.6)	20 (7.0)
Do you use toothpicks?	171 (60.2)	92 (32.4)	21 (7.4)
Do you use mouth rinse?	1 (0.4)	264 (93.0)	19 (6.7)
Do you clean with other things?	205 (72.2)	2 (0.7)	77 (27.1)
Do you have toothpaste in your home?	221 (77.8)	60 (21.1)	3 (1.1)
Do you use toothpaste?	230 (80.1)	53 (18.7)	1 (0.4)
Do you use toothpaste with fluoride? *	217 (95.4)	54 (19.0)	2 (0.7)

* Eleven (3.9%) participants replied “do not know” to the question, “Do you use toothpaste with fluoride”?
